# Public Health Risk Communication by Text Message in Response to a Cluster of Invasive Meningococcal Infection in a Primary School

**DOI:** 10.1371/currents.outbreaks.ba688e481f19515a472d3adfdb5143f8

**Published:** 2014-05-19

**Authors:** Declan T Bradley, Jillian Johnston, Brian Smyth

**Affiliations:** Public Health Agency, Belfast, Northern Ireland; Centre for Public Health, School of Medicine, Dentistry and Biomedical Sciences, Queen's University Belfast, Belfast, Northern Ireland; Public Health Agency, Belfast, Northern Ireland; Public Health Agency, Belfast, Northern Ireland

**Keywords:** meningococcal disease, risk communication, SMS text message

## Abstract

Public health risk communication during emergencies should be rapid and accurate in order to allow the audience to take steps to prevent adverse outcomes. Delays to official communications may cause unnecessary anxiety due to uncertainty or inaccurate information circulating within the at-risk group. Modern electronic communications present opportunities for rapid, targeted public health risk communication. We present a case report of a cluster of invasive meningococcal disease in a primary school in which we used the school’s mass short message service (SMS) text message system to inform parents and guardians of pupils about the incident, to tell them that chemoprophylaxis would be offered to all pupils and staff, and to advise them when to attend the school to obtain further information and antibiotics. Following notification to public health on a Saturday, an incident team met on Sunday, sent the SMS messages that afternoon, and administered chemoprophyaxis to 93% of 404 pupils on Monday. The use of mass SMS messages enabled rapid communication from an official source and greatly aided the public health response to the cluster.

## Introduction

Invasive infection with *Neisseria meningitidis* is a significant public health problem in the United Kingdom because of the rapid disease progression and deaths that occur in a minority of people who are exposed to the bacterium, and because of the potential for clusters and outbreaks. Those who develop invasive infection typically do so 1-2 weeks following initial exposure to the bacteria, and exhibit rapidly emerging signs of severe illness that often result in death or disability.^1^^,^ 2

Taylor-Robinson et al. of the UK Health Protection (HPA; now part of Public Health England) reported an evaluation of a communication intervention to inform secondary-level pupils and parents about an outbreak of meningococcal disease and to arrange mass antibiotic prophylaxis for all pupils and staff.^3^ Their outbreak became apparent on a Friday afternoon, and on the following Monday the HPA issued letters to parents and released a press statement for radio. Mass distribution of antibiotics was carried out on the Tuesday morning. In qualitative responses to a survey, many pupils reported that the official communications had been too slow, because interpersonal communication had spread the news over the preceding days, but that this had been accompanied by rumour, misinformation and uncertainty. The report indicated that 74% of school pupils already knew about the meningococcal disease outbreak before information was released by the school and the Health Protection Agency, partly because of the sharing of information by pupils using social media and text messages. Pupils' questionnaire responses suggested that a reliable source should have notified pupils and parents more quickly.^3^

Crisis and emergency risk communication is “The need to inform and alert the public about an event” in an emergency context.^4^ There is a likelihood of high emotional engagement (or “outrage”^5^) during crisis communications and communication must be “timely, accurate, direct and relevant.”^6^ Short Message Service (SMS) text messages have been used in emergency risk communication settings to inform and advise targeted members of the public. For example, during a large outbreak of conjunctivitis in Taiwan, infection control advice was disseminated by text message to parents of all school children in a city as part of a wider strategy to control the outbreak.^7^

We present a short case report of the management of a cluster of invasive meningococcal disease. The emphasis of the report is on communication methods, so that we can share with other health protection professionals the lessons that we learned when communicating with staff and students of an entire school by SMS text message to inform them and to arrange mass chemoprophylaxis.

## Case Report

In June 2012, the Public Health Agency (PHA) was notified of two children from different year groups in the same primary school who were being treated for meningococcal disease. Both children were assessed as meeting the Health Protection Agency guidelines case definition of 'probable' meningococcal disease following discussion between the attending paediatricians and public health physicians. The definition of 'probable' meningococcal disease is "[c]linical diagnosis of meningitis or septicaemia where... meningococcal disease is the most likely diagnosis".^8^ The first child developed symptoms on a Monday and was admitted to hospital the following day, at which time the PHA was notified. Real-time PCR investigation of a throat swab for meningococcal *ctrA *gene was reported to be positive, indicating carriage of or infection with the meningococcus, and strengthening confidence in the diagnosis of invasive meningococcal disease. The PHA conducted the usual public health follow-up of this individual case, including sending a letter to parents through the school providing information about meningococcal disease. The second child developed symptoms on the Friday and was admitted to hospital that night. Notification to the PHA occurred on Saturday morning. The family of the second patient reported to hospital staff that they had received the letter from the PHA informing them of the first case of meningococcal infection. Detailed enquiry of the social, school and extra-curricular activities for the preceding fortnight for both children identified no common settings or activities other than the school. The identities of the children were not revealed to the other parents. PHA on-call health protection team convened and led an incident team comprising PHA, pharmacy, community nursing and school representatives, which met on the Sunday morning in the school premises. The team followed national health protection guidance for such clusters^8^ and decided that all pupils and staff should receive chemoprophylaxis to reduce the risk of further cases occurring. The incident team planned how antibiotics would be offered to all pupils and staff the next day (Monday). The school was due to close three days later for the end of the summer term.

The primary school is one of seven in a medium-sized town of approximately 10,000 population. The super output area in which the school was located (population approximately 2,000 in the 2011 census) had a median age of 41 years and less than 1% of the population reported that English was not their first language. The school had 406 pupils and 52 permanent employees. The school subscribed to a commercial mass SMS text messaging service, which was used for communicating important information to parents.

Responding to the event presented significant communication and logistical challenges. These included: arrangements for informing parents and obtaining consent; collecting and recording information on those receiving antibiotic prophylaxis, to be prescribed under a Patient Group Directive and administered by Trust school nurses; securing supplies and transport of antibiotics, spoons and other equipment; informing the Primary Care Out of Hours Centres, A&E and paediatric units; as well as an email alert for all GPs; and preparing media briefings with professional public relations support from within the PHA. The incident team planned the flow of children and parents through school rooms to facilitate orderly information collection, assessment and administration of antibiotics.

Parents and guardians of pupils at the affected school were contacted through the school’s mass SMS mobile phone text message system on the Sunday afternoon at 1600 with a series of three messages. The messages explained that two children had been diagnosed with probable meningococcal disease and that antibiotics would be offered to all staff and pupils, that there may be press coverage and indicated times that parents and guardians should attend (Table 1). The messaging service did not allow recipients of the messages to respond by text message.

**Table 1. Mass SMS Text Messages:** SMS text messages were sent to guardians of pupils on Sunday afternoon informing them of the incident and giving information about the response.

4:00 PM	A 2nd pupil has meningococcal disease. Risk to other children low but if unwell see GP. Antibiotics arranged for tomorrow for all pupils. Details to follow.
4:02 PM	Antibiotics are precautionary. May be in news tonight. schedule for antibiotics to follow. www.teachers2parents.co.uk
4:05 PM	Children to attend as normal. Parents P1&2 attend at 1200 for child consent. Parents P3&4 at2pm, parents P5-7 at 3pm. Docs&nurses present to answer questions.

On Monday morning, parents were met at the school entrance and given a letter, consent form, antibiotic information leaflet and meningococcal disease information leaflet. Question and answer briefing sessions were held to share information with parents, who were noted to be concerned but calm. By the end of Monday (within 24 hours of the SMS text message communication), 376 (93%) of 404 pupils (excluding the two affected children) and all 52 permanent staff received prophylactic antibiotics. A second session was held on Tuesday, at which a further 11 pupils and 5 additional temporary staff received antibiotics. By the end of the second session 387 (96%) of the pupils had received chemoprophylaxis in school. A clinician was present during the antibiotic dispensing session to give advice if nursing staff raised a query about whether a child should receive antibiotics. Most children and staff received a single dose of ciprofloxacin, though seven pupils (2% of the pupil population) with ciprofloxacin contraindications received a two day course of rifampicin. Those who received antibiotics were observed for 30 minutes by school staff, who were briefed to alert the medical team in the event of adverse effects. There were no anaphylactic or allergic reactions, and one pupil reported nausea. Of the remaining pupils who did not receive prophylaxis in school, four received antibiotics from GPs, and five were on holiday and received antibiotics abroad or on return from GPs. Only four pupils could not be contacted by the time that the incident was considered closed one week after the first antibiotic dispensing session in the school. Four other children did not receive chemoprophylaxis due to receiving treatment for coincidental illnesses. The school informed PHA that the SMS system delivery report showed that all except one family received the SMS text message, and that family was contacted by telephone and the child received chemoprophylaxis in school. Informal feedback from parents to PHA staff indicated that they were, in general, happy with the content and method of communication.

On the day after mass antibiotic prophylaxis (eight days after the first case became symptomatic), a third child from same the locality, but from another school, was notified to the PHA as a probable case of meningococcal disease. All three children were of different ages. Detailed enquiry revealed no overlapping social or extra-curricular activities.

The first child to be affected had a positive real-time PCR test for meningococcal *ctrA* gene from a throat swab, negative blood cultures, and a positive blood real-time PCR test from the reference laboratory for *ctrA. *The second and third children had the same pattern of results (positive throat swab, negative blood cultures, positive blood PCR). The *siaD *PCR indicated serogroup B infection for all three cases. *PorA* sequence type for all three cases were identical.

## Discussion

The school’s access to a mass SMS system and up-to-date records of phone numbers was extremely valuable in allowing rapid communication with parents and guardians. The message came directly from the school, which meant that parents received information from a known, trusted source. This is likely to have pre-empted some of the rumour and misinformation described in the Taylor-Robinson et al. report.^3^ The resulting local and regional media coverage of the event was measured in tone, and the school reported to PHA that parents and guardians had been positive about the response to the incident.

SMS text messages offer a powerful way of delivering public health risk information to a specific audience, whilst providing a high degree of confidence that the message is likely to be read soon. In this setting, it presents some advantages over alternative methods of emergency risk communication, such as telephoning people individually (which is very resource-intensive), email (which is likely to be picked up less rapidly), and using the broadcast media (which, by introducing third parties into the chain of communication might risk increasing anxiety within and beyond the target audience). Communicating accurate and unambiguous information to parents and guardians within the 160 character limit of SMS messages was a novel challenge. We overcame this by sending a series of three text messages, each of which was coherent on its own but which built on the information from the previous message.

New social media (for example, Facebook and Twitter) would probably not have been as effective in reaching parents and guardians of primary school children. However, in other situations where names and telephone numbers were not available or were less reliable, social media might be a very useful tool for risk communication.
